# Raman Spectroscopy of Janus MoSSe Monolayer Polymorph Modifications Using Density Functional Theory

**DOI:** 10.3390/ma15113988

**Published:** 2022-06-03

**Authors:** Aleksandr S. Oreshonkov, Ekaterina V. Sukhanova, Zakhar I. Popov

**Affiliations:** 1 Laboratory of Acoustic Microscopy, Emanuel Institute of Biochemical Physics of Russian Academy of Sciences, 119334 Moscow, Russia; yekaterina.sukhanova@phystech.edu (E.V.S.); zipcool@bk.ru (Z.I.P.); 2 Laboratory of Molecular Spectroscopy, Kirensky Institute of Physics, Federal Research Center KSC SB RAS, 660036 Krasnoyarsk, Russia; 3 School of Engineering and Construction, Siberian Federal University, 660041 Krasnoyarsk, Russia

**Keywords:** MoSSe, dichalcogenides, Janus structure, Raman, polymorph, monolayer, DFT

## Abstract

Two-dimensional transition metal dichalcogenides (TMDs) with Janus structures are attracting increasing attention due to their emerging superior properties in breaking vertical mirror symmetry when compared to conventional TMDs, which can be beneficial in fields such as piezoelectricity and photocatalysis. The structural investigations of such materials, along with other 2D materials, can be successfully carried out using the Raman spectroscopy method. One of the key elements in such research is the theoretical spectrum, which may assist in the interpretation of experimental data. In this work, the simulated Raman spectrum of 1H-MoSSe and the predicted Raman spectra for 1T, 1T’, and 1H’ polymorph modifications of MoSSe monolayers were characterized in detail with DFT calculations. The interpretation of spectral profiles was made based on the analysis of the lattice dynamics and partial phonon density of states. The presented theoretical data open the possibility of an accurate study of MoSSe polymorphs, including the control of the synthesized material quality and the characterization of samples containing a mixture of polymorphs.

## 1. Introduction

Photocatalytic water splitting is considered one of the promising ways to solve global energy and environmental issues [1] for many reasons, among which are the ability to organize large-scale production and practically unlimited resources (water and solar light), which seems to be the most attractive. The conversion of solar energy into chemical energy in nature occurs through the photosynthesis process, and the process of artificial (industrial) photosynthesis involves biomimicry of this natural phenomenon. The main goal of the artificial photosynthesis process is to obtain pure hydrogen energy (H_2_) [2]. The direct decomposition of water by visible light irradiation is impossible. However, a photoelectrochemical method using a semiconductor (for example, TiO_2_) as an absorber of solar radiation was proposed by A. Fujishima and K. Honda in 1972 [3]. Since then, a significant number of compounds such as g-C_3_N_4_ [4], ZnO [5], porous metal–organic frameworks (MOFs) [6], GaP [7], CdS [8], and other compounds [9] have been proposed for use in semiconductor photocatalysis. Nanosized materials can also be used as catalyst materials; for example, there are several works related to the study of the possible application of SnS_2_/g-C_3_N_4_ heterostructures [10], ZnO nanowires [11], TiO_2_/SnO_2_ nanofibers [12], 2D MXenes [13], CdS nanoparticles [8], MoS_2_ nanosheets [14], etc.

Recently, new types of two-dimensional monolayers, the so-called Janus structures, were proposed as promising nanomaterials in photocatalytic water-splitting reactions [15,16]. The main feature of these nanomaterials is the presence of two inequivalent surfaces, leading to the appearance of additional dipole moments in the transverse direction [17]. For example, the Janus MoSSe structure consists of three layers of atoms: sulfur, molybdenum, and selenium [18]. Such a monolayer structure is assumed to be an efficient wide solar-spectrum photocatalyst material for water-splitting reactions [19,20], with characteristics that are superior to currently known 3D and 2D materials. After the successful synthesis of a 1H (honeycomb, graphene-like structure) MoSSe monolayer in 2017 [21], several polymorph phases have been investigated theoretically: 1T [22], 1T’ [23], and 1H’ [24] monolayers, and even MoSSe nanotubes [25].

The Raman spectroscopy method provides a fast and accurate determination of the structural characteristics of bulk and nanosized materials and makes it possible to distinguish different structures. This method was successfully used to investigate several 2D nanomaterials, for example, graphene [26,27], MoS_2_ [28,29], WS_2_ [30], MoSe_2_ [31], WSe_2_ [31,32], black phosphorus monolayer [33], etc. Experimentally fabricated 1H MoSSe Janus monolayers were successfully characterized in the terms of Raman spectroscopy, and Raman spectra were obtained at room [34,35,36] and cryogenic [34] temperatures. It should be noted that the experimental data interpretation can be facilitated by comparing the experimental and theoretically predicted Raman spectra.

In this paper, we present the DFT calculation of Raman spectra with a detailed interpretation of vibrations for different polymorph modifications of Janus MoSSe monolayers. The calculated spectrum of the 1H phase shows an excellent agreement with known experimental data; thus, the selected computational model and parameters can be used for the prediction of Raman spectra of MoSSe polymorphs not yet experimentally synthesized. In turn, predicted spectra can be used for both the experimental identification of individual phases in multiphase samples and quality control of monolayer structures.

## 2. Calculation Details

All calculations were performed within the framework of density functional theory (DFT [37]), using local density approximation [38] based on the Perdew and Zunger parametrization [39] of the numerical results of Ceperley and Alder [40], as implemented in the CASTEP code [41]. Norm-conserving pseudopotentials were used, and the plane-wave cutoff was set to 720 eV. The reciprocal space was sampled by 7 × 7 × 1, 7 × 7 × 1, 4 × 7 × 1, and 3 × 3 × 1 Monkhorst–Pack k-meshes for 1H, 1T, 1T’, and 1H’ phases of MoSSe, respectively. Monolayers were relaxed until the maximum forces became smaller than 0.03 eV/Å, and the maximum stress was less than 0.05 GPa. The convergence criterion of the self-consistent minimization of total energy was set to be equal to 1 × 10^−7^ eV. The size of the vacuum slab for the different phases was no less than 17.5 Å. A visualization of the monolayer structure was performed within VESTA software [42]. The Raman spectrum for each phase was simulated by a Lorentzian broadening, equal to 10 cm^−1^.

## 3. Results and Discussion

### 3.1. 1H Modification

**Figure 1:**
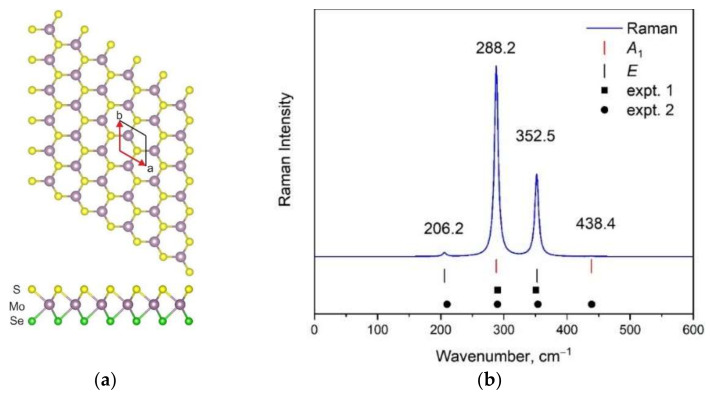
Top and side views of 1H MoSSe modification (**a**) and corresponding simulated Raman spectra (**b**). Calculated wavenumbers are shown by vertical lines. Experimental wavenumbers were taken from [18] (expt. 1) and [34] (expt. 2).

The atomic structure of the hexagonal 1H MoSSe monolayer [21] is presented in Figure 1a. In this and the next figures, purple-, yellow-, and green-colored balls represent molybdenum, sulfur, and selenium atoms, respectively. The unit cell consists of three atoms that result in nine normal vibrational modes in the center of the Brillouin zone (BZ). The 1H modification is characterized by the space group *P*3*m*1 (*C*_3v_) in the Hermann–Mauguin (Schoenflies) notation, and the vibrational representation can be written as Γ_vibr_(*C*_3v_) = 3*A*_1_ + 3*E*. All vibrational modes are Raman and infrared (IR) active, and *A*_1_ and *E* modes are acoustical. The calculated Raman spectrum of this modification is shown in Figure 1b. The spectral profile is characterized by one strong, one medium, and two very weak bands. The intensities of Raman bands and their positions are in total agreement with experimental data obtained in some studies [18,34,43]. To date, the DFT-calculated wavenumber values for the 1H phase are presented in several works [34,43,44], and the simulated spectral contour can be also found in the literature [43]. The accuracy of results obtained in this work (both wavenumber values and Raman intensities) is no worse, and in some cases even better, than that presented in the literature. Thus, the chosen theoretical model can be applied for the prediction of Raman spectra of other MoSSe phases.

### 3.2. 1T Modification

**Figure 2:**
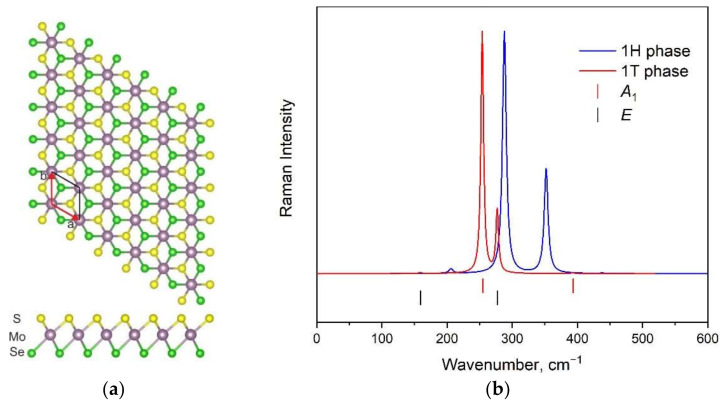
The structure of 1T MoSSe modification (**a**). The comparison of calculated Raman spectra for 1H and 1T-MoSSe modifications (**b**). Calculated wavenumbers for 1T phase are shown by vertical lines.

The displacement of one atomic layer (S or Se) in the 1H MoSSe monolayer leads to the formation of a new modification called 1T (see Figure 2a). Nevertheless, the number of atoms in the unit cell remains the same as in the 1H phase; thus, the number of vibrational modes should also be unchanged. The simulated Raman spectrum of the 1T phase is compared with the Raman spectrum of the 1H phase in Figure 2b. A significant shift in the band positions is observed.

**Figure 3:**
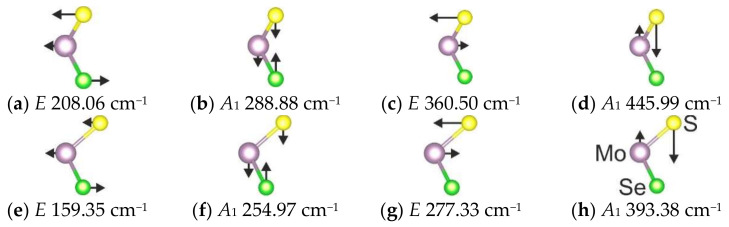
A schematic representation of vibrational modes of 1H (**a**–**d**) and 1T (**e**–**h**) MoSSe modifications.

In Figure 3, the atomic displacements (eigenvectors) of vibrational modes for 1H and 1T modifications are depicted. The non-degenerate *A*_1_ modes correspond to an out-of-plane atomic movement, while the doubly degenerate *E* modes are in-plane vibrations. The largest shift in wavenumbers between the 1H and 1T phases is observed in the case of spectral bands with medium intensity (see Figure 2b). These bands correspond to in-plane Mo-S translations, and such behavior is associated with a significant difference in Mo-S band length in 1H and 1T modifications.

**Figure 4:**
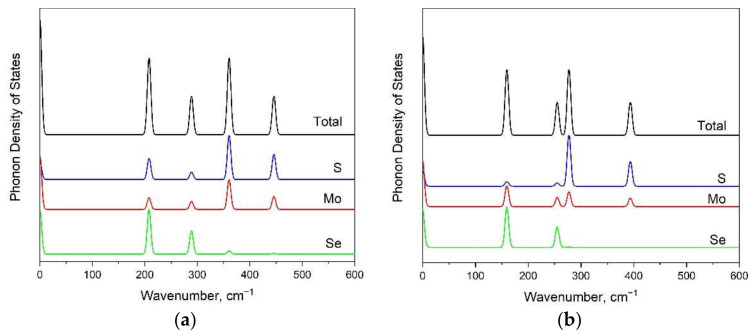
Total and partial phonon densities of states for 1H (**a**) and 1T (**b**) MoSSe monolayers.

From the analysis of phonon density of states (Figure 4), we can infer that Se-related vibrations are dominant in low-wavenumber regions: below 290 cm^−1^ for the 1H phase, and below 260 cm^−1^ for the 1T phase (see Figure 4a,b, respectively). The molybdenum-involved vibrations are in all wavenumber ranges, and the sulfur translations are strong in the high-wavenumber range.

### 3.3. 1T’ Modification

**Figure 5:**
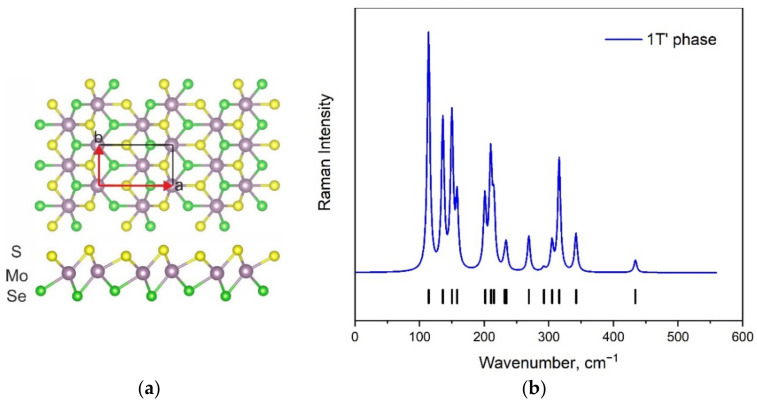
The structure of 1T’-MoSSe modification (**a**). Simulated Raman spectrum of phase (**b**). Calculated wavenumbers are shown by vertical lines.

The deformed 1T structure is denoted as the 1T’ phase [23,45], and its atomic structure is presented in Figure 5a. Since the 1T’ modification is described by the *P*1 (*C*_1_) space group, the irreducible representations of all normal modes will be labeled as *A*. Six atoms in the unit cell result in eighteen normal vibrational modes in the center of the BZ, and three of these modes are acoustical. Thus, we can conclude that fifteen spectral lines can be observed in Raman spectra of 1T’-MoSSe modification. The calculated Raman spectrum of this modification is shown in Figure 5b, and a significant difference is observed in comparison with the 1H and 1T phases.

**Figure 6:**
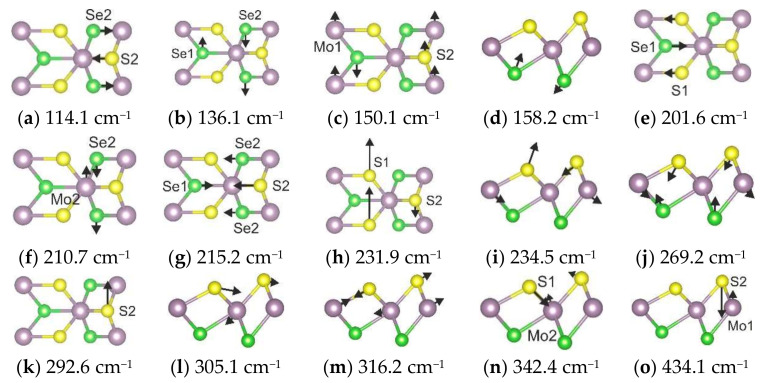
Schematic representations of the eigenvectors corresponding to the calculated vibrational modes of 1T’-MoSSe modification.

The high-intensity spectral bands below 155 cm^−1^ (Figure 5b) are in-plane vibrations of S/Se, Se, and S/Mo/Se, as shown in Figure 6a–c, respectively. A medium spectral band at 158 cm^−1^ is an antiphase, out-of-plane translation of Se atoms, shown in Figure 6d. The left shoulder of the peak at 210 cm^−1^ is a S/Se in-plane vibration, the peak itself is a Se/Mo in-plane translation, and the right shoulder of this peak is related to the S/Se in-plane translations (see Figure 6e–g, respectively). A weak band at 234 cm^−1^ in Figure 5b is related to antiphase translations of sulfur, as shown in Figure 6h,i. A single weak band at 269 cm^−1^ is associated with out-of-plane S-Mo-Se stretching (Figure 6j). Weak bands at 292 and 305 cm^−1^ are in-plane Mo-S vibrations, as shown in Figure 6k,l. The intense peak at 316 cm^−1^ is related to the antiphase Mo-S stretching vibrations (Figure 6m) and is a combination of out-of-plane and in-plane atomic vibrations. The band related to S-Mo vibration is located at 342 cm^−1^ (see Figure 6n). Although the calculated Raman spectra of the 1T’ phase are more complicated than the spectra of the 1H and 1T phases, a very weak spectral band is observed at the highest wavenumber region, identical to the 1H modification, and it is related to S-Mo vibration (Figure 6o). A large shift in wavenumbers of S-Mo vibrations in the 1T’ phase is associated with a difference in S2-Mo1 and S1-Mo2 bond lengths.

**Figure 7:**
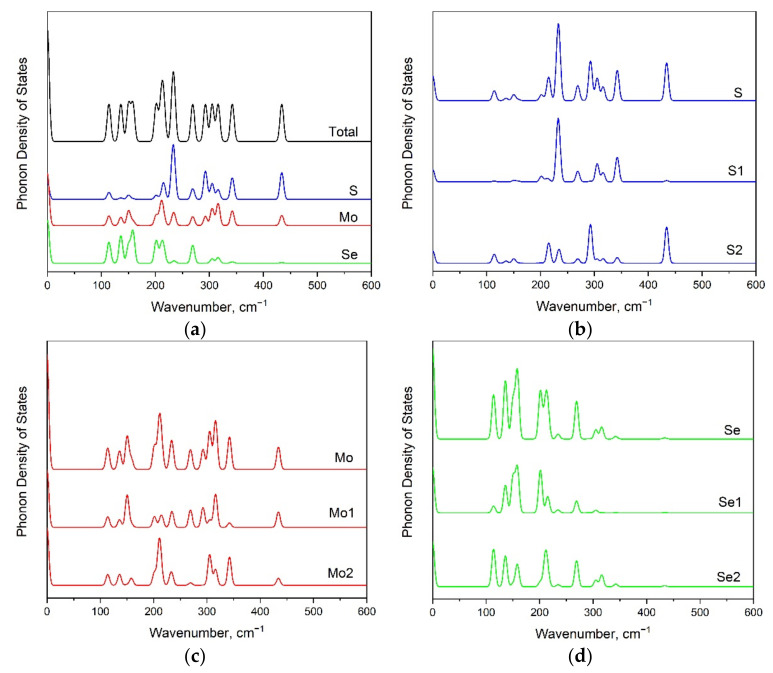
The phonon density of states for 1T’-MoSSe modification (**a**) including partial contributions of each S (**b**), Mo (**c**), and Se (**d**) ions.

In order to clarify the impact of individual atoms on a particular spectral line, the phonon density of states is presented in Figure 7a. The significant contribution from S vibrational modes to phonon density of states is observed above 205 cm^−1^ (see Figure 7b). The Mo atoms participate in vibrational modes in various regions of the vibrational spectrum (see Figure 7c). The phonon density of states below 330 cm^−1^ involves Se translations (see Figure 7d).

### 3.4. 1H’ Modification

**Figure 8:**
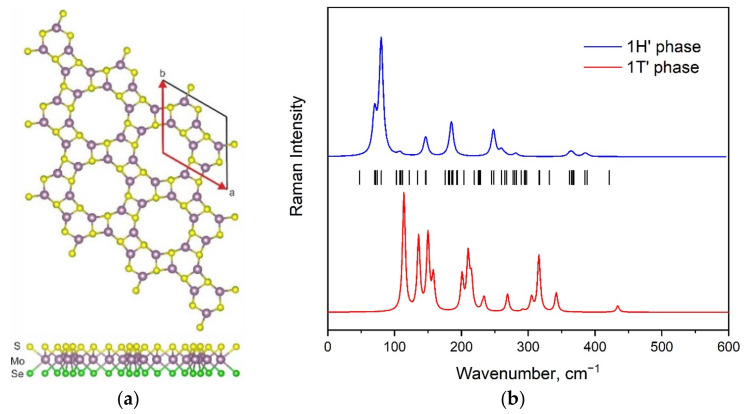
The structure of 1H’-MoSSe modification (**a**). Simulated Raman spectrum of this phase compared with Raman spectrum of 1T’ modification (**b**). Calculated wavenumbers are shown by vertical lines.

The 1H’ phase of transition metal dichalcogenides was first described in the work of Y. Ma et al. [24], and the atomic structure of 1H’-MoSSe is presented in Figure 8a (space group *P*1 (*C*_1_)). The total number of atoms in the conventional cell of this modification is equal to 18. This results in 54 vibrations, of which 3 are acoustical while other modes are optical. Figure 8b presents the calculated Raman spectrum of the 1H’-MoSSe modification compared with the calculated Raman spectrum of the 1T’ phase, and a shift in intense peaks toward lower wavenumbers is observed.

**Figure 9:**
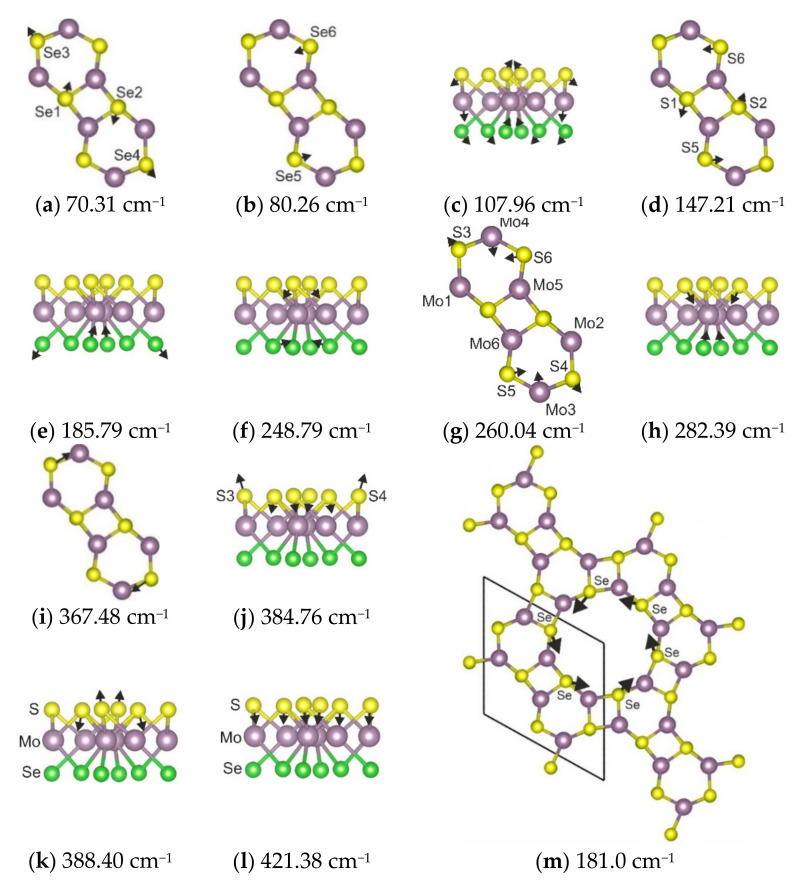
Eigenmodes of 1H’ MoSSe selected vibrations.

Due to a large number of Raman-active vibrational modes, description is given for only the prominent Raman peaks. A graphical representation of these modes is shown in Figure 9.

The spectral profile of the 1H’ phase contains strong peaks at 80 cm^−1^ and medium peaks at 70, 147, 185, and 248 cm^−1^. Several weak bands are observed at 108, 260, 282, 364, and 386 cm^−1^. The medium band at 70 cm^−1^ is an in-plane Se translation, shown in Figure 9a. The strong band at 80 cm^−1^ is also related to in-plane Se vibrations (Figure 9b), but it involves a different set of atoms compared to the previous spectral band. All types of atoms involved in the vibrations appear as a weak band at 108 cm^−1^ (Figure 9c). The medium band at 147 cm^−1^ is a S in-plane vibration, as presented in Figure 9d. The medium-intensity band at 185 cm^−1^ is related to a Se out-of-plane vibration, as shown in Figure 9e. The medium band at 248 cm^−1^ is a combination of S out-of-plane and Se in-plane vibrations (Figure 9f). The weak band at 260 cm^−1^ is the S and Mo in-plane vibration, seen in Figure 9g. The weak spectral band at 282 cm^−1^ is a S-Mo-Se stretching-like vibration (Figure 9h). The next weak band, at 364 cm^−1^, is related to the in-plane vibration of sulfur ions (Figure 9i). The weak band at 386 cm^−1^ is an antiphase, out-of-plane S-Mo vibration, as shown in Figure 9j,k. The vibrational mode with the highest eigenfrequency is related to the S-Mo out-of-plane stretching, as shown in Figure 9l. It is interesting to note that some of the vibrational modes can be represented by considering neighboring crystallographic cells. For example, in the case of vibration with a wavenumber equal to 180 cm^−1^ (Figure 9m).

**Figure 10:**
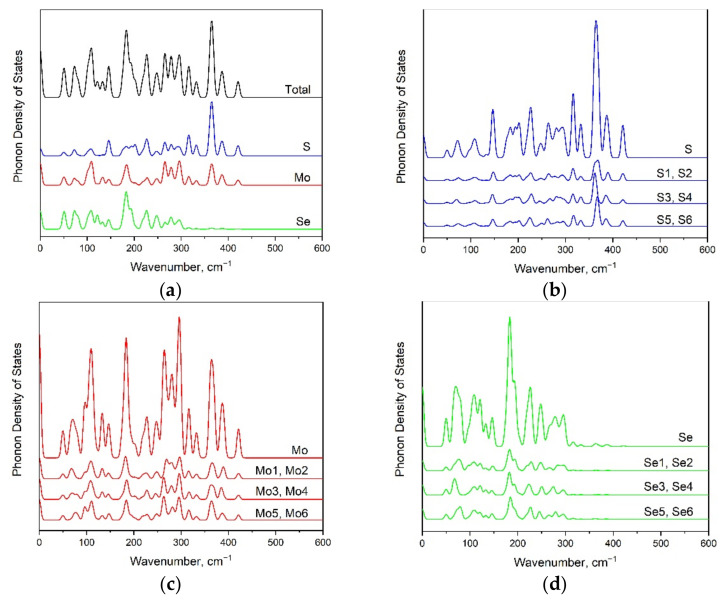
Total (**a**) and partial (**b**–**d**) phonon density of states for 1H’ MoSSe monolayer.

The calculated phonon density of states for the 1H’ modification of a MoSSe monolayer is presented in Figure 10. The partial phonon DOS clearly illustrates that both sulfur and molybdenum atomic vibrations contribute to the whole spectral range. The selenium vibrations are below 300 cm^−1^. Figure 10 shows that partial phonon density states of atoms are identical for pairs of atoms, such as S1 and S2, S3 and S4, S5 and S6, Mo1 and Mo2, etc.

## 4. Conclusions

The Raman spectra for 1H, 1T, 1T’, and 1H’ phases of a MoSSe monolayer were successfully simulated using the density functional theory approach. The observed spectral bands were assigned to corresponding vibrations in all spectral regions, and the contribution of individual atoms to the spectrum obtained was estimated using calculations of partial phonon density of states. It was shown that the form of the spectral profile was similar for 1H and 1T modifications, but a significant shift in the positions of spectral lines was observed. The simulated spectra of 1T’ and 1H’ phases were significantly richer in lines in comparison with the honeycomb modifications of MoSSe. Nevertheless, the Raman spectra of these two phases were easily distinguishable, both by the number of intense bands and by the range of the spectra. Thus, the spectral range of the 1T’ phase began at 100 cm^−1^ but at 50 cm^−1^ in the case of the 1H’ phase. This feature is explained by the difference in the atomic structures of these phases, which once again demonstrates the advantage of using the Raman spectroscopy method to study 2D materials. Finally, the results of this work can be used for the study of certain phases and for their quality control.
